# Male fertility versus sterility, cytotype, and DNA quantitative variation in seed production in diploid and tetraploid sea lavenders (*Limonium* sp., *Plumbaginaceae*) reveal diversity in reproduction modes

**DOI:** 10.1007/s00497-012-0199-y

**Published:** 2012-10-20

**Authors:** Ana Sofia Róis, Generosa Teixeira, Timothy F. Sharbel, Jörg Fuchs, Sérgio Martins, Dalila Espírito-Santo, Ana D. Caperta

**Affiliations:** 1 Plant Diversity and Conservation Group, Centro de Botânica Aplicada à Agricultura (CBAA), Instituto Superior de Agronomia, Technical University of Lisbon, Tapada da Ajuda, 1349-017 Lisbon, Portugal; 2 Faculdade de Farmácia, Centro de Biologia Ambiental (CBA), University of Lisbon, Avenida Professor Gama Pinto, 1649-003 Lisbon, Portugal; 3 Apomixis Research Group, Department of Cytogenetics and Genome Analysis, Leibniz Institute of Plant Genetics and Crop Plant Research (IPK), 06466 Gatersleben, Germany; 4 Karyotype Evolution Group, Department of Cytogenetics and Genome Analysis, Leibniz Institute of Plant Genetics and Crop Plant Research (IPK), 06466 Gatersleben, Germany; 5 Centro de Ecologia Aplicada “Baeta Neves”, Instituto Superior de Agronomia, Technical University of Lisbon, Tapada da Ajuda, 1349-017 Lisbon, Portugal

**Keywords:** Apomixis, Genome size, *Limonium*, Male sporogenesis, Male gametogenesis, Polyploidy

## Abstract

The genus *Limonium* Miller, a complex taxonomic group, comprises annuals and perennials that can produce sexual and/or asexual seeds (apomixis). In this study, we used diverse cytogenetic and cytometric approaches to analyze male sporogenesis and gametogenesis for characterizing male reproductive output on seed production in *Limonium ovalifolium* and *Limonium multiflorum*. We showed here that the first species is mostly composed of diploid cytotypes with 2*n* = 16 chromosomes and the latter species by tetraploid cytotypes with 2*n* = 32, 34, 35, 36 chromosomes and had a genome roughly twice as big as the former one. In both species, euploid and aneuploid cytotypes with large metacentric chromosomes having decondensed interstitial sites were found within and among populations, possibly involved in chromosomal reconstructions. *L. ovalifolium* diploids showed regular meiosis resulting in normal tetrads, while diverse chromosome pairing and segregation irregularities leading to the formation of abnormal meiotic products are found in balanced and non-balanced *L. multiflorum* tetraploids. Before anther dehiscence, the characteristic unicellular, bicellular, or tricellular pollen grains showing the typical *Limonium* micro- or macro-reticulate exine ornamentation patterns were observed in *L. ovalifolium* using scanning electron microscopy. Most of these grains were viable and able to produce pollen tubes in vitro. In both balanced and unbalanced *L. multiflorum* tetraploids, microspores only developed until the “ring-vacuolate stage” with a collapsed morphology without the typical exine patterns, pointing to a sporophytic defect. These microspores were unviable and therefore never germinated in vitro. *L. ovalifolium* individuals presented larger pollen grains than those of *L. multiflorum*, indicating that pollen size and ploidy levels are not correlated in the *Limonium* system. Cytohistological studies in mature seeds from both species revealed that an embryo and a residual endosperm were present in each seed. Flow cytometric seed screens using such mature seeds showed quantitative variations in seeds ploidy level. It is concluded that male function seems to play an important role in the reproduction modes of *Limonium* diploids and tetraploids.

**Electronic supplementary material:**

The online version of this article (doi:10.1007/s00497-012-0199-y) contains supplementary material, which is available to authorized users.

## Introduction

The genus *Limonium* Miller (sea lavenders) is the most species-rich and widespread of the *Plumbaginaceae* and comprises halophytes inhabiting sea shores, salt marshes, and salt steppes (Erben 1993). For this genus, a reticulate diversification model involving changes in ploidy levels has been proposed and is thought to be responsible for its high taxonomic complexity (Palacios et al. 2000; Lledó et al. 2005). Several systematic and phylogenetic studies tried to clarify the taxonomic complexity of this genus in a global perspective (Baker 1948; Pignatti 1971; Karis 2004; Lledó et al. 2005) or specifically on particular geographic areas (Ingrouille 1984; Artelari 1989; Artelari and Georgiou 2002). A large number of microspecies have been described especially in the Mediterranean basin (Erben 1993). Among them, it has been accepted that truly sexual species are infrequent, whereas it is assumed that facultative apomictic species account for a large proportion of the species (Erben 1978; Cowan et al. 1998), although this is yet to be confirmed. Taxonomic complexity has been linked to a sporophyte self-incompatibility pollen–stigma dimorphism system (Baker 1966) and the polyploid hybrids ability to produce seeds by apomixis (asexual reproduction by seed) (D’Amato 1949; Baker 1953a, b, 1966; Erben 1978). In members of the *Limonium* genus, reduced and unreduced embryo sacs have been described. In addition to tetrasporic 8-nucleate *Fritillaria*-type or *Adoxa*-type, or tetrasporic 16-nucleate *Penea*-type female gametophytes (Dahlgren 1916; D’Amato 1940), *Ixeris*-type embryo sacs with non-haploid eggs are found in triploid (2*n* = 3*x* = 27) *Statice oleaefolia* var. *confusa* (the present genus *Limonium* was known as *Statice*; *Statice*, *nom. rej*. vs. *Armeria*; Greuter et al. 2000) (D’Amato 1949). In this last type of megasporogenesis, the first meiotic division ends with the formation of a restitution nucleus followed by a second meiotic division not accompanied by cytokinesis (meiotic diplospory), followed by two further mitotic divisions of the unreduced nuclei to result in 8-nucleate embryo sacs.

Various studies have explored the cytological variability of *Limonium* species from several regions and have reported diploid (2*n* = 2*x* = 16, 18), triploid (2*n* = 3*x* = 24, 25, 27), tetraploid (2*n* = 4*x* = 32, 35, 36), pentaploid (2*n* = 5*x* = 43), and hexaploid (2*n* = 6*x* = 51, 54, 56) species (Erben 1978; Brullo and Pavone 1981; Arrigoni and Diana 1993; Castro and Rosseló 2007). Diploid species (2*n* = 16 or 2*n* = 18) seem to be apparently stable, with typically two basic chromosome numbers, *x* = 8 and *x* = 9 (Erben 1978). It has been hypothesized that triploid *Limonium* are predominant in the genus, presumably arisen through hybridization (allopolyploids) of two diploid types with basic chromosome numbers *x* = 8 and *x* = 9 which supply the resulting hybrids with reduced and unreduced gametes (Erben 1978, 1979). In polyploid species, different chromosome numbers have been shown within the same species, population or even in the same specimen (Dolcher and Pignatti 1967, 1971; Diana 1995; Castro and Rosseló 2007). The most extreme karyological polymorphism was reported in the Corsican endemic *L. bonifaciense*, where in more than 50 % of the seedlings mixoploidy occurred (Diana 1995). However, knowledge on interspecific, intraspecific, and population level variability in species of the South–West Atlantic Coast is limited.

Palynological studies in *Limonium* have revealed the two *Armeria* pollen grain types with an exine surface coarsely prominently reticulate (A-pollen) or finely reticulate (B-pollen), and pollen sizes between 40 and 100 μm (Erdtman 1952; Nowicke and Skvarla 1977). In *Limonium,* the sporophytic self-incompatibility system is linked with pollen–stigma dimorphism. A-pollen type grains germinate on papillose stigmas and B-pollen type germinate in *cob*-like stigmas, while the complementary combinations produce no successful fertilization (Baker 1953a, b; Richards 1997). In general, pollen stainability appears to be high in sexual diploid, while in polyploids, low to high fertility has been reported (Erben 1978, 1979). For example, in the hexaploid *L. humile* (2*n* = 6*x* = 54), a high percentage of fertile pollen grains is found (95 %) (Dawson and Ingrouille 1995), while in the triploid *L. viciosoi* (2*n* = 3*x* = 27), pollen was either not produced or with very low stainability (2–13 %) (Erben 1978). Although a few studies exist in male gametophyte development in vitro in *Limonium* horticultural valuable crops (ex. *Limonium perezii*) (Zhang et al. 1997), to our knowledge, detailed studies on male sporogenesis and gametogenesis are lacking.

In the South–West Iberian Peninsula shorelines, about 15 *Limonium* species have been recognized (Erben 1993, 1999). Among them, the species of the *L. ovalifolium* complex group which show marked morphological similarities and have been described as sexual diploids (2*n* = 2*x* = 16) are represented by *L. ovalifolium* (Poir.) O. Kuntze, *L. nydeggeri* Erben and *L. lanceolatum* (Hoffmanns & Link) Franco (Erben 1993, 1999). The Lusitania highly endemic aneuploid tetraploid *L. multiflorum* Erben (2*n* = 4*x* = 35), presumably an apomict, is considered as a crop wild relative from mainland Portugal (Brehm et al. 2008). This species belongs to the *L. binervosum* complex group which also includes *L. binervosum* (G.E.Sm.) C.E. Salmon and *L. dodartii* (Girard) Kuntze (Erben 1978). However, till now studies within these groups were based on limited sampling of taxa and on morphological studies, and knowledge in their reproductive modes is lacking.

The aim of the present study is to characterize male sporogenesis and gametogenesis to determine male reproductive output in seed production in diploid *L. ovalifolium* and polyploid *L. multiflorum*. To this end, we first studied the ploidy levels through genome size measurements and chromosome counts in plants from both species. Then, we analyzed male sporogenesis and gametogenesis using optical and scanning electron microscopy. To reconstruct the mode of reproduction within these groups, we analyzed mature seeds by means of a flow cytometric seed screen procedure, cytohistology, and electron scanning microscopy. Our results show contrasting male fertility versus sterility in both species, likely associated with distinct reproductive strategies.

## Materials and methods

### Plant materials and growth conditions

**Fig. 1:**
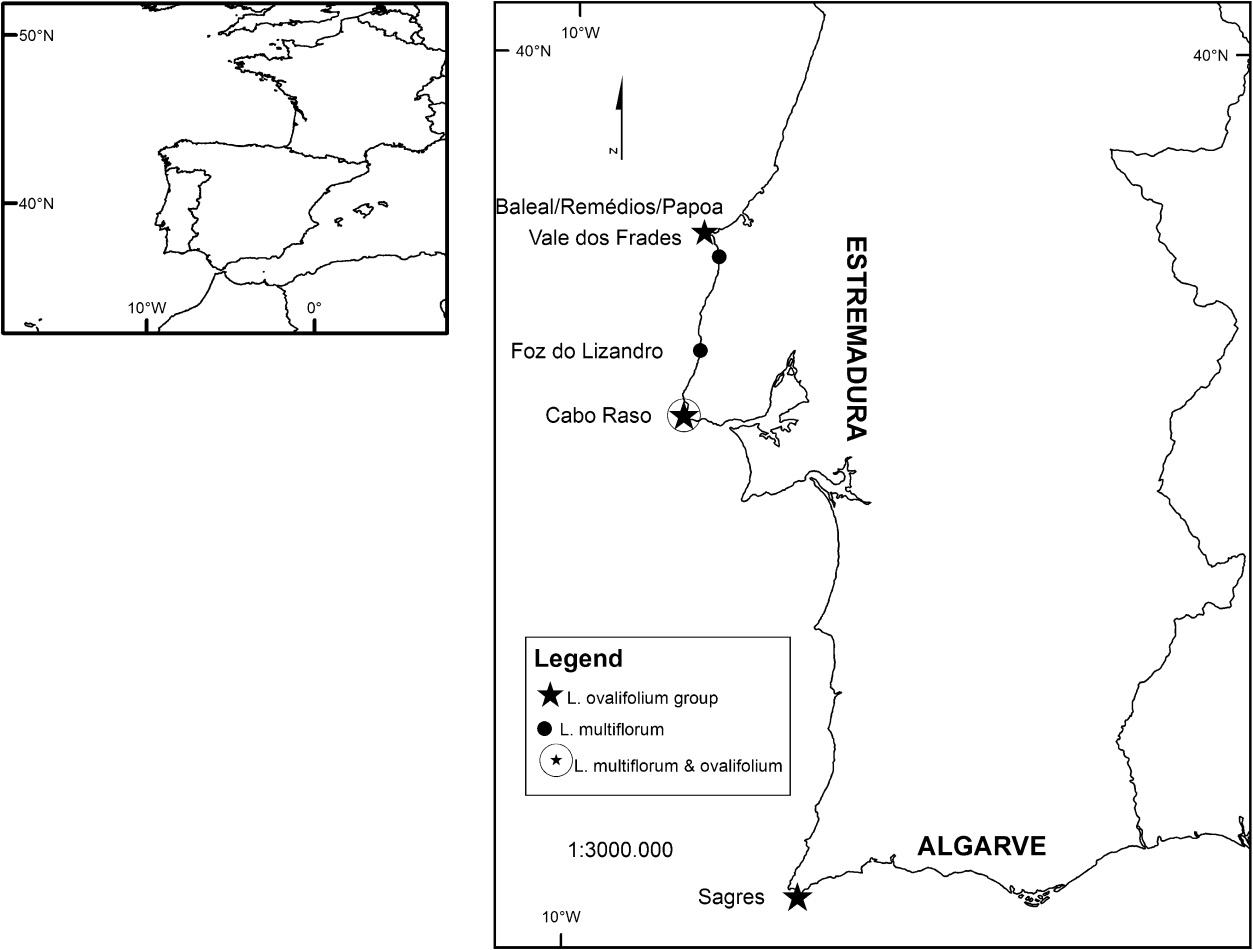
*Limonium ovalifolium* and *L. multiflorum* populations sampled in continental Portugal from Estremadura (Baleal, Papoa, Sra dos Remédios, Vale dos Frades, Foz do Lizandro, Cabo Raso) and Algarve (Sagres) provinces

The species were identified using keys from Erben (1993), and herbarium specimens from the Herbarium João de Carvalho e Vasconcelos, LISI, were examined to confirm species identifications. Fieldwork was carried out in the Estremadura, Southwest Alentejo, and Algarve Provinces. For each species, seeds were collected in the wild from up to twenty specimens from each population. In this work, we will refer to *L. ovalifolium* in sensu lato to the sampled populations from Baleal (B) (Estremadura: Peniche), Papoa (P) (Estremadura: Peniche), Srª Remédios (SR) (Estremadura: Peniche), Cabo Raso (CR) (Estremadura: Cascais), and Cabo de Sagres (CS) (Algarve: Sagres). *L. multiflorum* populations were sampled from Vale dos Frades (VF) (Estremadura: Lourinhã), Foz do Lizandro (FL) (Estremadura: Ericeira), and Cabo Raso (Estremadura: Cascais) (Fig. 1). All populations were tagged with Global Positioning System, and mapping was made using ArcGIS Desktop 10 (ESRI).

To establish controlled experimental populations from both species, about fifty seeds per population from five individuals randomly selected were placed on moist filter paper in Petri dishes and then transferred to a growth chamber (Rumed) for germination with controlled light and temperature with a photoperiod of 18 h light and 6 h dark at 25 °C until germination. The germinating seeds were transplanted into plastic pots with substrate and grown under greenhouse conditions, prior to use in cytological studies. Species identifications were confirmed with particular emphasis placed on leaf, inflorescence, and flower morphology on 8-month-old plants.

### Chromosome preparation and karyotyping

Seven distinct plants from each species population growing in the greenhouse were randomly chosen and analyzed. Root tips were excised and then treated with a 2 mM 8-hydroxyquinoline solution for 2 h at 4 °C in the dark and subsequently for 2 h at room temperature to induce metaphases. Then, root tips were fixed in a fresh absolute ethanol:glacial acetic acid (3:1) solution overnight and stored at −20 °C until used. Next, root tips were digested with a 2 % cellulase, 2 % cellulase “Onozuka R-10”, and 2 % pectinase enzyme solution in 1xEB (40 ml 0,1 M citric acid-1-hydrate and 60 ml of 0,1 M sodium citrate dihydrate; pH 4,8) for 3 h at 37 °C as described in Caperta et al. (2008). Squashes were made in 45 % acetic acid, and preparations were counterstained with 4′,6-diamidino-2-phenylindole hydrochloride (DAPI) (1 mg/ml) diluted in Citifluor (Agar).

### Genome size estimations

For flow cytometric genome size estimations, roughly 10 mm^2^ of leaf tissue from individuals of *Limonium* populations was chopped with a sharp razor blade together with leaf material of either *Pisum sativum* L. subsp. *sativum* convar. *sativum* var. *ponderosum* Alef., Sorte Viktoria, Kifejtö Borsó, (2*C* = 9.07 pg) (Genebank Gatersleben accession number: PIS 630) or *Secale cereale* L. subsp. *cereale*, (2*C* = 16,01 pg) (Genebank Gatersleben accession number:Sortiments-Nr. R 737) as internal reference standards in a Petri dish containing 1 ml nuclei isolation buffer (Galbraith et al. 1983) supplemented with 1 % PVP-25, 0.1 % Triton X-100, DNase-free RNase (50 μg/ml) and propidium iodide (50 μg/ml). The nuclei suspension was filtered through a 35-μm mesh cell strainer cap and stored on ice until measurement. The relative fluorescence intensities of stained nuclei were measured using a FACStar^PLUS^ (BD Biosciences, New Jersey, USA) flow sorter equipped with an argon ion laser INNOVA 90C (Coherent, Palo Alto, CA, USA). Usually, 10,000 nuclei per sample were analyzed. The absolute DNA amounts of samples were calculated based on the values of the G1 peak means.

### Cytological analyses of microsporogenesis

Flower buds of distinct floral stages were collected before anthesis from three diploid *2n* = 16 *L. ovalifolium* individuals and three polyploid *L. multiflorum* individuals, respectively, 2*n* = 32, 35, and 36 growing in the greenhouse. Anthers were dissected and fixed in a fresh absolute ethanol:glacial acetic acid (3:1) solution. The material was digested with 2 % cellulase, 2 % cellulase “Onozuka R-10”, and 2 % pectinase enzyme solution in 1xEB for 45 min at 37 °C. Squashes were made in 45 % glacial acetic acid according to Caperta et al. (2008). The preparations were counterstained with DAPI.

### In vitro pollen viability tests

The fluorescent diacetate (FDA) test procedure was used to determine pollen viability as described in Heslop-Harrison and Heslop-Harrison (1970), with some modifications. In brief, FDA was made up as a stock solution 2 mg/ml of acetone. Immediately before use, dilutions were prepared by adding drops of the stock to 2 ml of sucrose solution. The working solution prepared according to this procedure gave a concentration of 6 × 10^−5^ M FDA in 0.5 M sucrose (Pinillos and Cuevas 2008). All pollen grains, which fluoresced brightly, were scored as viable. Total viability estimates were performed by one person using one to seven flowers per plant and counted under 20× magnification. For *L.ovalifolium,* 500 pollen grains per flower were recorded, while for *L. multiflorum* depending on the availability up to 250 pollen grains per flower were counted.

### In vitro pollen germination

Three flowers (five anthers each flower) per plant in five replicates were used to analyze pollen tube growth. The pollen grains were collected from plants soon after anther dehiscence and were then cultured in a media containing 20 mM boric acid, 6 mM calcium nitrate, 0.1 % CH and 7 % sucrose (Zhang et al. 1997). A dialysis tubing and filter paper support combined with using 23 % polyethylene glycol −20,000 as an osmoticum in the medium provided appropriate physical conditions for pollen germination. Pollen grains were incubated at 37 °C during 48 h or 72 h in the dark. Pollen grains were considered germinated when they had a tube length that was equal or greater than the diameter of the pollen grain. For measurement of tube length, 40 pollen tubes were selected randomly from each treatment and measured on micrographs recorded with a 63× objective using the Axiovision 4.0 (Zeiss).

### Optical microscopy analysis and imaging

Cell preparations, meiocytes, pollen grains, and pollen tubes were observed using a Zeiss Axioskop 2 fluorescence microscope. Images were collected with an AxioCam MRc5 digital camera (Zeiss) and further processed using Adobe Photoshop 5.0 (Adobe Systems, Mountain View, CA, USA). Well spread chromosome complements were measured on micrographs recorded with a 63× objective using the Axiovision 4.0 (Zeiss).

### Electron microscopy pollen and seed analyses

Flower and seed samples of both species were fixed in a 2.5 % glutaraldehyde solution in 0.1 M sodium phosphate buffer, pH 7.2, for 5 h at 4 °C as described in Hayat (1981). Then, the material was dehydrated in a graded ethanol series, from 30, 50, 75, and 100 % ethanol/water for 30 min each. Flowers were then dried at a critical point on a Critical Point Polaron BioRad E3500, and coated with a thin layer of gold on a Jeol JFC-1200. Observations were carried out on a scanning electron microscope JSM-5220 LV (SEM) equipped with a direct image acquisition system. To estimate pollen morphology and dimensions, randomly chosen grains were measured from SEM micrographs. Pollen terminology and dimensions followed that of Erdtman (1952). Pollen grain dimensions were based on the length of the longest pollen grain axis, and four dimension classes were considered, including very small (*x* ≤ 10 μm), small (10 > *x* ≤25 μm), medium (25 > *x* ≤50 μm), and large (50 > *x* ≤100 μm). Mean, standard deviation, and standard error of the mean were estimated.

### Cytohistological analysis of seeds

To show the cellular structure of the different tissues of mature seeds, they were fixed as described above for flower samples and embedded in paraffin following standard methods (Ruzin 1999). Longitudinal sections were then cut on a Leitz 1512 Minot microtome, at 10–12 μm and stained with Lugol’s solution for starch localization (Johansen 1940). Cytohistological observations were made with a Nikon Labophot 2 light microscope (LM) using 10× and 40× objectives, and images were obtained with a Nikon FX-35 W camera.

### Flow cytometric screening of seeds

For flow cytometric seed screening (FCSS, Matzk et al. 2000), at least seven specimens per population collected in the wild and fifty mature seeds per plant were used. Single seeds were ground individually with three 2.3-mm stainless steel beads in each well of a 96-deep-well plate containing 80 μl isolation buffer (OTTO1) using a Geno-Grinder 2000 (SPEX CertiPrep) at a rate of 50 strokes/min for 2 min (Matzk et al. 2000). Afterward 200 μl of isolation buffer was added to recover enough volume for filtration (30 μm mesh). 50 μl of the resultant suspension was stained by adding 150 μl of staining buffer (OTTO2 + 1–2 μg/ml DAPI) and incubated on ice for 10 min before flow cytometric analysis. All sample plates were analyzed at 4 °C on a temperature-regulated Robby-well auto-sampler hooked up to a Partec PAII flow cytometer (Partec GmbH, Munster, Germany). To validate the data, five seeds per plant per accession were analyzed with a conventional razor-chopping method according to (Matzk et al. 2000). Histograms representing the quantification of nuclei with distinct DNA content were plotted using a linear scale (x-axis).

## Results

In both, *L. ovalifolium* and *L. multiflorum,* estimations of seed set per inflorescence from different individuals of the analyzed populations were higher than a hundred seeds. The overall germination rate was slightly higher in *L. multiflorum* (mean 68 %) than in *L. ovalifolium* (mean 63 %), although intraspecific differences between populations were detected. In *L. ovalifolium,* the germination rate ranged from 30 % in P to 77 % in CS populations, while in *L. multiflorum,* it varied from 70 % in CR to 80 % in FL populations.

**Fig. 2:**
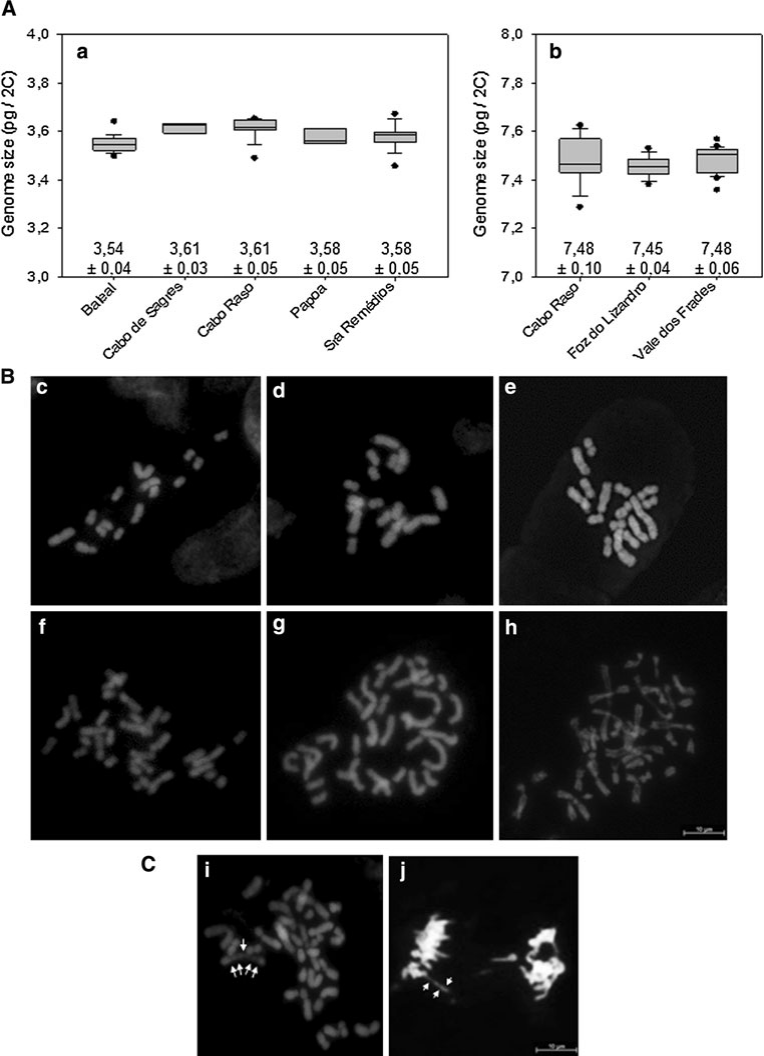
**A** Boxplots showing technical replicate distribution of genome size measurements for different localities of *L. ovalifolium* (**a**) and *L. multiflorum* (**b**). For each locality, three to eighteen individuals were analyzed. **B** Mitotic metaphase plates of DAPI-stained chromosome spreads from seven distinct individuals of different *Limonium ovalifolium* (**c**–**e**) and *L. multiflorum* (**f**–**h**) populations. **c** Baleal, 2*n* = 15; **d** Cabo Raso, 2*n* = 16; **e** Sagres, 2*n* = 16; **f** Cabo Raso 2*n* = 32; **g** Vale Frades 2*n* = 35; **h** Foz do Lizandro 2*n* = 36; **C** Cabo Raso 2*n* = 36 mitotic cells showing a large metacentric chromosome with insterstitial decondensation sites (*arrowed*) in metaphase (**i**) or anaphase (**j**). The fluorescent images have been digitally inverted

The absolute genome size of diploid *L. ovalifolium* and tetraploid *L. multiflorum* was found to be 3.58 ± 0.04 pg/2C and 7.47 ± 0.07 pg/2C, respectively. While the values of genome size in distinct populations were quite similar in *L. multiflorum* (FL: 7.45 ± 0.04 pg/2C; VF: 7.48 ± 0.06 pg/2C; CR: 7.48 ± 0.1 pg/2C), they showed a slightly higher variability in *L. ovalifolium* and ranged from 3.54 pg/2C in the B population to 3.61 pg/2C in the CS and CR populations (Fig. 2a–b). However, co-processing of individuals from B together with individuals of either CS or CR populations did not result in double peaks as would be expected to confirm distinct intraspecific genome size variation.

Karyotype analysis and chromosome counts were made on microphotographs of mitotic metaphase spreads of five *L. ovalifolium* and ten *L. multiflorum* specimens from each population. In *L. ovalifolium,* most specimens showed 2*n* = 16 chromosomes (Fig. 2d, e), with a pair of large metacentric chromosomes and four pairs of small metacentric chromosomes that measured 5.5–7 μm and 1.4–2.5 μm, respectively, and three pairs of submetacentric chromosomes that measured 2.5–4 μm. Nonetheless, aneuploid cytotypes with 2*n* = 15 (Fig. 2c) or 2*n* = 17 chromosomes also appeared especially in the B population. With regard to *L. multiflorum*, in all populations, we found individuals with 2*n* = 32, 34, 35, or 36 chromosomes (Fig. 2f–h). This karyological diversity was also present within cells of a few specimens rendering a precise count virtually impossible. With regard to chromosome types, we observed one or two large metacentric chromosomes measuring 5–9 μm, four medium metacentric chromosome pairs ranging 3–8 μm, three large submetacentric chromosome pairs measuring 6–10 μm, seven medium submetacentric chromosome pairs 3–5.9 μm, and variable numbers of small metacentric or telocentric chromosomes which measured less than 2.9 μm. Occasionally, three to five interstitial constrictions could be observed on the large metacentric chromosomes (Fig. 2i).

Calculations of total chromosome lengths (μm) in ten cells with approximately the same degree of condensation revealed for both species that metaphase cells with a higher chromosome numbers measured also higher chromosome length. For example, in *L. multiflorum,* total chromosome length in cells with 2*n* = 34 chromosomes was 104,5 μm, while those with 2*n* = 35 was 114.0 μm and with 2*n* = 36 was 194.0 μm. Furthermore, measurements of chromosome length of the large metacentric chromosomes in 25 metaphase cells from each species showed that in *L. ovalifolium,* two large metacentric chromosomes were always present, whereas in *L. multiflorum,* one or two large metacentric chromosomes could be found. Nine metaphase cells displayed size differences between the large metaphase chromosomes of around 2 μm, which corresponds to the average size of a telocentric chromosome. Sixteen metaphase cells showed only one large metacentric chromosome. However, independent of the presence of one or two large metaphase chromosomes, we detected cells with 2*n* = 34, 35, or 36 chromosomes. Although this demonstrates that at least in *L. multiflorum,* the large metacentric chromosome might be involved in chromosomal reconstruction, it does not explain all variable cytotypes.

**Fig. 3:**
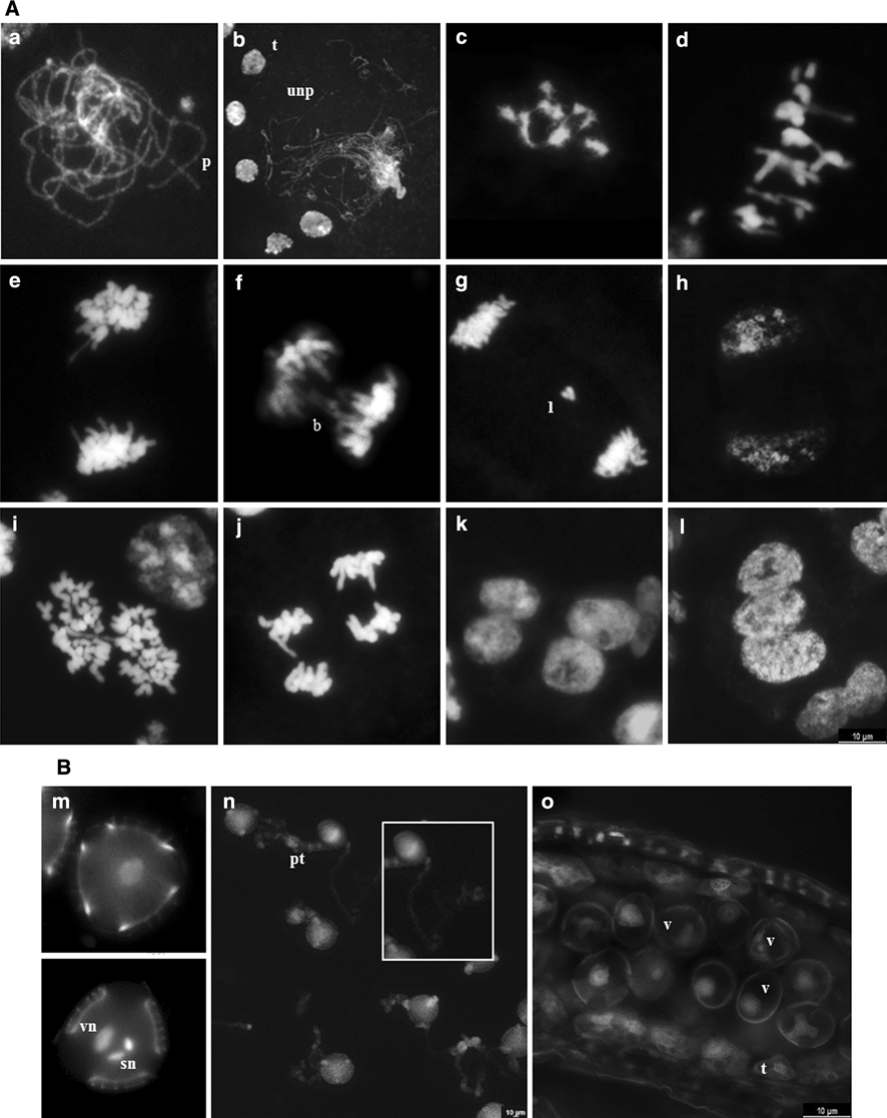
Chromosome pairing and segregation in DAPI-stained male sporocytes (**A**) and male gametogenesis (**B**) in *Limonium ovalifolium* and *L. multiflorum*. **A** Full pairing (p) of chromosomes at pachytene in *L. ovalifolium* (**a**); Incomplete pairing (unp) of chromosomes at pachytene in *L. multiflorum*; compare meiocytes with small *tapetum* cells (t) (**b)**. Diakinesis/metaphase I transition in *L. ovalifolium* showing bivalents (**c**); Metaphase I in *L. multiflorum* with univalents and bivalents (**d**); **e**–**g** *L. multiflorum* anaphase I meiocytes showing balanced segregation (**e**, **f**) chromosome bridges (**b**) and lagged chromosomes (l) (**g**); **h** Balanced telophase I in *L. multiflorum*; **i** *L. multiflorum* meiocytes in metaphase/anaphase transition during second meiotic division; **j** *L. ovalifolium* meiocytes in anaphase II; **k** *L. ovalifolium* normal tetrad; **l** *L.multiflorum* triad; **B** *L.* *ovalifolium* unicellular pollen (**m**, *upper image*) and tricellular pollen grain with a vegetative nucleus (vn) and two sperm nuclei (sn) (**m**; *lower image*); **n** *L. ovalifolium* pollen tubes (pt) germinated in vitro; the *inset* exhibits a detailed pollen tube; **o** Longitudinal section of a non-dehisced anther from *L. multiflorum* showing unicellular microspores exhibiting a large vacuole (v) causing the characteristic “signet-ring” appearance; the *tapetum* cells are in the periphery of the anther (t). The fluorescent images have been digitally inverted

Next, we analyzed male meiosis in both species, although it was difficult to select anthers of identical developmental stages for comparative investigation since flower and anther dehiscence appears to occur earlier in *L. multiflorum* than in *L. ovalifolium*. In this latter, one homologous chromosome pairing at pachytene seemed to be complete (Fig. 3a), contrasting to that of the tetraploid *L. multiflorum* where some chromosomes were unpaired (Fig. 3b). In *L. ovalifolium*, eight sets of homologs were visible at diplotene and congressed during diakinesis/metaphase I transition (Fig. 3c). In *L. multiflorum,* metaphase I plates showed bivalent and univalent chromosomes (Fig. 3d), which either exhibited an apparent balanced segregation at anaphase I (Fig. 3e) resulting in well-formed dyads (Fig. 3h) or presented several segregation anomalies such as peculiar chromosome bridges (Fig. 3f) or anaphase lag chromosomes (Fig. 3g). In some anaphase I cells from both species, we were able to detect the large chromosome with interstitial constrictions (Fig. 2j). In both species, second division meiocytes were found (Fig. 3i–j), and at the end of telophase II, normal tetrads or triads were visible in *L. ovalifolium* (Fig. 3k), whereas normal and abnormal tetrads, triads, or polyads were seen in *L.multiflorum* (Fig. 3l). Before anther dehiscence, unicellular pollen, bicellular or tricellular pollen grains were observed in *L. ovalifolium*, the latter with the characteristic vegetative nucleus and two sperm nuclei (Fig. 3m). In this species, about 93 % of the pollen grains were viable and germinated in vitro, forming pollen tubes with approximately 33–274 μm in length (Fig. 3n). Instead, in both balanced and unbalanced *L. multiflorum* tetraploids, the majority of pollen grains only attained the “ring-vacuolate” microspore stage (Stage 8; Owen and Makaroff 1995) in which microspores exhibited a large vacuole causing the characteristic “signet-ring” appearance (Fig. 3o). Although about 37 % of pollen grains were viable, pollen tubes were never observed, even not after 72 h of incubation in the germination medium.

**Fig. 4:**
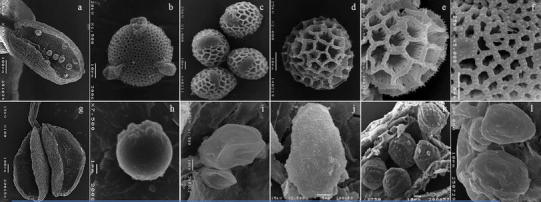
Scanning electron microscopy photographs of *Limonium ovalifolium* (**a**–**f**) and *L. multiflorum* (**g**–**l**) pollen grains. **a** Dehisced anther with a few pollen grains still inside; **b** Spheroid tricolpate pollen grain with microreticulate exine surface (*polar view*); **c** Ellipsoid grains with macroreticulate exine surface (*diverse equatorial views*); **d** Pollen grain with coarsely reticulate exine surface with a large *reticulum* (*polar view*); **d** Detail of the irregular macroreticulate exine net with spinose side walls; **f** Detail of the *reticulum* with strait spaces in microreticulate exine surface; **g** Dehisced anther without pollen grains; **h** Radiosymetric pollen grain with psilate exine ornamentation; **i** Bilateral pollen grains with distinct dimensions; **j** Bilateral pollen grains showing perforate exine surface with no prominent spines; **k** Pollen grains with verrucate or warty exine surface with *colpi* position denounced; **l** Pollen grain with perforate exine surface and with *colpi* position denounced

Examination of SEM micrographs of anthers and pollen grains revealed totally different patterns between species. In general, *L. ovalifolium* pollen grains could still be seen within the anthers (about 100 each anther) (Fig. 4a). These grains were spheroidal in the polar view, while in the equatorial outline, they tended to be ellipsoid, isopolar, radiosymetric, and tricolpate, with usually long *colpi*, acute in the apices, and with a smooth membrane (Fig. 4b, c). Two ornamental patterns in exine surface could be seen, macroreticulate (Fig. 4c, d) or microreticulate (Fig. 4b). In the first type, a large and irregular *reticulum* with spinose side walls (*muri* spinulose) (Fig. 4e) and regular *columellae* fused distally into an incomplete *tectum* was observed (Fig. 4d, e). Pollen grains with microreticulate exine surface with a tighter *reticulum* and less spinulose *muri* were only observed in plants from CS population (Fig. 4b, f). Most plants showed pollen grains of medium size (Table 1) and, except for B population, a certain percentage of large pollen grains (Table 1).

**Table 1:** *Limonium ovalifolium* and *L. multiflorum* pollen grain dimensions (μm) according to the classes of Erdtman (1952)

Species	Population	Mean	stdev*	% Grains in dimension classes**				Number of grains analyzed
				≤10	10 > *x* ≤24	25 > *x* ≤49	50 > *x* ≤100	
*L. ovalifolium*	Baleal	40.43	3.01	0	0	100	0	35
	Cabo Raso	46.79	6.18	0	0	86	14	44
	Cabo Sagres	42.69	5.29	0	0	92	8	71
	Papoa	45.14	3.51	0	0	95	5	39
	Sra. Remédios	45.65	3.14	0	0	92	8	36
*L. multiflorum*	Cabo Raso	30.6	11.2	8	16	73	3	38
	Foz do Lizandro	21.77	8.39	0	27	73	0	30
	Vale dos Frades	29.34	4.28	0	11	89	0	36

* stdev Standard deviation

** Dimension Classes (μm) according to Erdtman (1952): very small (*x* ≤ 10); small (10 > *x* ≤24); medium (25 > *x* ≤49); big (50 > *x* ≤100)

In contrast, *L. multiflorum* SEM micrographs revealed several empty anthers (Fig. 4g) or presented few pollen grains (<100), with diverse morphology and sizes. Most of the pollen grains were collapsed in morphology among different flowers of the same plant. A very small proportion, however, appeared regular in form (Fig. 4h), although lacking the typical exine ornamentation described for the *Limonium* genus (Erdtman 1952; Nowicke and Skvarla 1977). Whenever it was possible to determine pollen grain symmetry, isopolar, radiosymetric (Fig. 4h), and bilateral grains were detected (Fig. 4i–l). A highly variable exine surface, almost smooth or psilate (Fig. 4h, i), perforate or retipilate, with no prominent spines (Fig. 4j, l), verrucate or warty (Fig. 4k, l), was found, and the *colpi* were not visible but their position could be denounced (Fig. 4k). On average, pollen grains from *L. multiflorum* were smaller than those of *L. ovalifolium* (Table 1). CR individuals showed the highest variability in pollen grains size, from very small to large grains (Table 1). Instead, VF and FL individuals only showed small and medium size grains (Table 1).

Small mature capsules of both species contained only a single seed of approximately 1.8 mm (±0.3) in length and 0.4 mm (±0.1) in width. The estimation of embryo and residual endosperm nuclear DNA contents by FCSS (Matzk et al. 2000) showed that in *L. ovalifolium* populations, only histograms with a single DNA peak, 2C (channel 50), 3C (channel 75) or 4C (channel 100) were found (supplemented Fig. S1) representing diploid or triploid seeds, respectively (Table 2). Neither a 3C endosperm peak, nor secondary peaks of (endo-) replicated endosperm nuclei were observed. Also, in *L. multiflorum,* only a single DNA peak was observed for each seed (supplemented Fig. S2), although variation in ploidy levels was obtained within the populations studied. For example, in *L. multiflorum* CR population, most specimens produced triploid or tetraploid seeds although other ploidy levels were found as well (Table 2).

**Table 2:** Summary of seed flow cytometric data with the observed ploidy levels in *Limonium ovalifolium* and *L. multiflorum* seeds

Species	Population	Percentage (%) of seeds C values							Number of individuals analyzed
		2C	3C	4C	5C	6C	7C	8C	
*L. ovalifolium*	Baleal	23	65	12	–	–	–	–	14
	Cabo Raso	78	22	–	–	–	–	–	12
	Cabo Sagres	79	20	–	–	–	–	–	16
	Lagos	–	–	–	–	–	–	–	0
	Papoa	–	–	–	–	–	–	–	0
	Sra. Remédios	54	44	2	–	–	–	–	14
*L. multiflorum*	Cabo Raso	6	50	30	–	6	7	1	13
	Foz do Lizandro	15	15	29	4	17	16	4	18
	Vale dos Frades	28	43	11	4	13	–	–	7

**Fig. 5:**
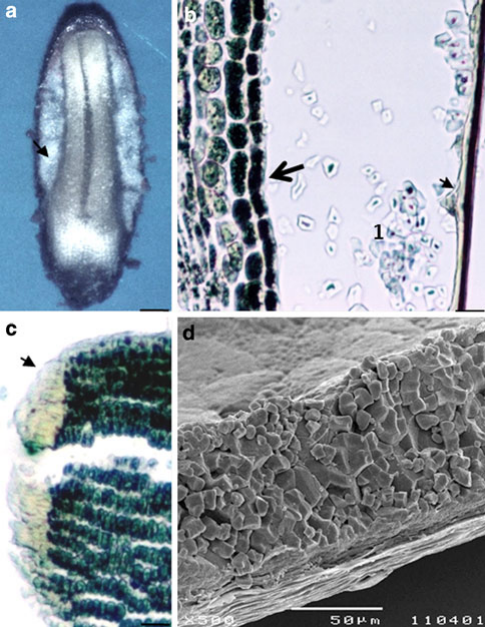
Structure and composition of *L. ovalifolium* and *Limonium multiflorum* and mature seeds. **a**–**b** *L. multiflorum*, LM micrographs. **a** View of a seed embedded in a paraffin block (*scale bar* 2 μm). **b** Detail of a longitudinal section, embryo tissue (*large arrow*) enveloped by a starchy endosperm (1) and a single aleurone layer (*small arrow*) covered by the seed coat is observed (*scale bar* 20 μm); **c** *L. ovalifolium*, LM micrograph showing the embryo tissue with a basal transfer layer (*arrow*) (*scale bar* 30 μm). **d** *L. ovalifolium*, SEM micrograph demonstrating a starchy endosperm with polygonal grains

Cytohistological and SEM studies of mature seeds from both species showed both an embryo and a residual endosperm, the latter being about 0.01 mm thick at the widest part in each seed (Fig. 5a). In both species, the embryo was enveloped by an endosperm tissue that no longer contained nuclei and was formed exclusively by polygonal starch grains with a well-defined hilum in the center, often with radiating clefts (Fig. 5b, d). The embryo was surrounded by an aleurone layer (7.0 μm (±0.2) thick) (Fig. 5b) and a basal transfer layer (45 μm (±8)) (Fig. 5c) surrounded by the seed coat. Therefore, it seems that the individual ploidy peaks observed in each seed corresponded to the embryo ploidy levels, since the residual endosperm did not contain any nuclei.

## Discussion

In this study on ploidy and chromosome variation, male sporogenesis and gametogenesis, pollen and seed analyses of *L. ovalifolium* and *L. multiflorum*, we have found differences in genome size and chromosome numbers between and within species, meiotic dysfunction, pollen variability in terms of morphology, size and fertility and quantitative variation in seed ploidy from single mother plants.

Genome size measurements in leaf material confirmed a diploid genome for *L. ovalifolium* and a tetraploid genome for *L. multiflorum* with a size roughly twice that of *L. ovalifolium*. No major differences in genome size were detectable within populations of each species. Previous data on *L. ovalifolium* chromosome numbers have shown that this *taxon* is composed of diploid cytotypes with 2*n* = 16 chromosomes, (Erben 1978, 1993), which is largely confirmed by the analysis of our samples, although we found a few aneuploid cytotypes. Also, we show here that both diploid and aneuploid diploid *L. ovalifolium* individuals present a peculiar, large metacentric chromosome pair which may present interstitial constrictions. Erben (1978) designated these chromosomes as marker chromosomes and showed that they were absent in species with 2*n* = 18 chromosomes. Conversely, in *L. multiflorum* specimens, in addition to unbalanced aneuploid tetraploids with 2*n* = 35 chromosomes (Erben 1978), we found also balanced cytotypes with 2*n* = 32, 34, and 36 chromosomes of different types. In both tetraploids cytotypes, one or two large metacentric chromosomes were present in which three to five interstitial constrictions were occasionally visible. Although our results provide evidence that the large metacentric chromosome could be involved in some kind of chromosomal reconstructions, it is not responsible for all aneuploidies observed. For example, in cytotypes with only one metacentric chromosome, we do not observe necessarily an increased number of small chromosomes as one could expect. Also, karyological studies in other *Limonium* species have revealed more than a single chromosome number and sometimes ploidy level for each species, population, or even within the same individual (Dolcher and Pignatti 1967, 1971; Arrigoni and Diana 1993; Diana 1995; Castro and Rosseló 2007). This was particularly emphasized for the polyploid *L. humile* in which different cytotypes were found among and within four populations (2*n* = 36, 38, 48, 49, 50, 51, 52, 54) (Dawson and Ingrouille 1995), and in the polyploid *L. carvalhoi* from the Balearic Islands in which three cytotypes were detected (2*n* = 24, 25, and 26) (Rosseló et al. 1998). This situation is also commonly observed in species-rich apomictic genera, for example, in *Boechera*, in which intraspecific ploidy polymorphisms have been reported for geographically widespread species (Kantama et al. 2007).

Cytological investigations of microsporogenesis in diploid (*2n* = 2*x* = 16) *L. ovalifolium* and in balanced (2*n* = 4*x* = 32 or 36) or unbalanced (2*n* = 4*x* = 35) *L. multiflorum* tetraploids have shown striking differences between species. In *L. ovalifolium,* meiosis essentially follows the regular course described for higher plants (Bhatt et al. 2001), whereby meiocytes at first prophase feature full pairing and normal chiasma formation with 8 bivalents at diakinesis and first metaphase followed by the reductional division of the homologous chromosomes. Meiotic figures characteristic for the equational division such as tetrads are also observed, indicating that reduced gametes are formed. Conversely, in *L. multiflorum* tetraploids, during the first prophase at pachytene, incomplete pairing is visible, and sometimes at the first metaphase, bivalents and univalents are seen. In the first anaphase, disturbances in segregating chromosomes such as chromosome bridges and/or laggard chromosomes are detected. Consequently, triads, normal and abnormal tetrads, and polyads are produced, likely leading abnormal microspores. Comparatively, in *Taraxacum officinale* and *Boechera holboelli* apomicts, incomplete pairing, chromosome association through stickiness, and unbalanced meiotic division have also been observed (Van Baarlen et al. 2000; Kantama et al. 2007).

**Fig. 6:**
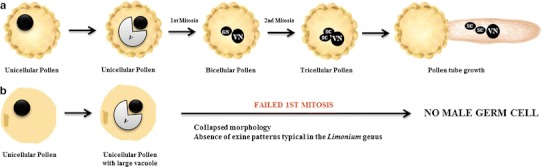
Diagrams for explaining pollen grain development in *L. ovalifolium* (**a**) and *L. multiflorum* (**b**). **a** In *L. ovalifolium* pollen grains follow a normal, first asymmetric mitotic division producing a generative cell (GC) within the vegetative pollen grain cell (VN) in the binucleate pollen stage. The second pollen division occurs before anther dehiscence and pollen germination originates tricellular pollen with two sperm cells (SCs). At this stage microspores are surrounded by the differentiated exine characteristic of the *Limonium* genus. When pollen germination occurs, the pollen tube growth is achieved. **b** In *L. multiflorum* unicellular pollen never undergoes first mitosis. Even after anther dehiscence, released pollen only attains the “ring-vacuolate” microspore with a large vacuole causing the characteristic “signet-ring” appearance (following the system of Owen and Makaroff 1995). The male germ unit is not produced. Key: *VN* vegetative nucleus, *GN* generative nucleus, *SC* sperm cells, *V* vacuole

In *L. ovalifolium*, after the tetrad stage, microspores undergo two mitotic divisions before anther dehiscence and pollination, resulting in the formation of tricellular pollen grains with a vegetative cell and two smaller sperm cells (McCue et al. 2011). Scanning electron microscope analyses reveal *Armeria* types A- and B-pollen, where the exine shows a distinct *reticulum* and pollen sizes between 40 and 100 μm (Erdtman 1952; Nowicke and Skvarla 1977). Most of these grains were viable as revealed by the FDA procedure tests and give rise to pollen tubes after germinating in vitro (Fig. 6a). Contrastingly, in *L. multiflorum* after the second meiotic division, only unicellular pollen grains are observed, even in dehisced anthers. These microspores only attained the stages 6–8 of pollen development, and some of them show the characteristic “signet-ring” appearance, in which the cytoplasm and nucleus are peripheral (Stage 8 of Owen and Makaroff 1995) (Fig. 6b). Furthermore, these grains which reveal a collapsed morphology and lack the typical exine patterns for *Limonium* species, never germinate in vitro. Thus, in addition to meiotic disturbances in pairing and segregation, we observed defects in pollen development and exine patterning in post-tetrad stages. Various studies of pollen fertility in *Limonium* polyploids, for example, in the triploid species *L. supinum* (2*n* = 3*x* = 26) and *L. viciosoi* (2*n* = 3*x* = 27), low pollen fertility has been reported (respectively, 3–27 % and 2–13 %) (Erben 1978). While in balanced tetraploids such as *L. vulgare* (2*n* = 4*x* = 36) or in the hexaploid *L. humile* (2*n* = 6*x* = 54), a high percentage of fertile pollen grains was detected (respectively, 96 and 95 %) (Dawson and Ingrouille 1995). In our case, however, both balanced and unbalanced tetraploids showed male sterility. Moreover, our results indicate for the first time that pollen size and ploidy are not correlated in the *Limonium* system, since in tetraploids most grains are smaller than in diploids. Considering that natural selection pressure on male gamete formation may be relaxed relative to female gamete formation in asexual *taxa* (Maynard-Smith 1978; Mogie et al. 2007; Voigt et al. 2007), the variability in pollen formation measured here and the absence of pollen tube formation in vitro are consistent with degeneration of the male function, possibly due to mutation accumulation.

Flow cytometric seed screening investigations have demonstrated the potential of this methodology to determine the routes of seed formation (Matzk et al. 2000). However, in both species, only one DNA peak was found which corresponds to the embryo peak, since mature seeds are characterized by one embryo and a well-developed starchy endosperm without nuclei. At an earlier stage of seed development, cells with nuclei might have been present. Nevertheless, it was not possible to get enough immature seeds for FCSS experiments due to their small size and the various layers of involucres in which they are enclosed (ovary, petals, calyx, inner, medium, and outer bracts). Hence, we cannot differentiate between an absence of endosperm peak in the FCSS analyses because of a low number of nucleated endosperm cells, or because a uninucleate central-cell (having same genome size as embryo) was produced. In previous works of other *Limonium* species female gametophytes of *Fritillaria*-type, *Adoxa*-type and *Penea*-type have been described (Dahlgren 1916; D’Amato 1940), and also non-reduced embryo sacs of *Ixeris*-type (D’Amato 1949). Moreover, we find quantitative variation for seed ploidy, and thus, if we attribute the single DNA peak to the embryo peak, this variation could be either due to fertilization of rare fertile pollen grains with varying ploidy. Nevertheless, after having tried to germinate *L. multiflorum* pollen grains several times, pollen tubes were never obtained. Alternatively, tetraploids could show partial apomixis via an uncoupling of apomeiosis and parthenogenesis in the seed material. Hence, ploidy shifts could be derived from female meiosis with parthenogenesis resulting in embryos with lower ploidies (polyhaploids), and/or apomeiosis combined with fertilization revealing higher ploidies, as in the so-called B_III_ hybrids (Matzk et al. 2000). In *Ranunculus kuepferi,* diploids resist introgression of apomixis, while among polyploids, only tetraploid apomicts form stable populations; the other cytotypes that arise by partial apomixis fail to establish (Cosendai and Hörandl 2010). Interestingly, even if a moderate to high germination ratio is observed in *L. ovalifolium*, none of the karyotyped seeds were triploid. Also, in *L. multiflorum,* only tetraploid seeds gave rise to plantlets, probably due to cytotype disadvantages.

In the sporophytic self-incompatibility system earlier described for *Limonium,* A-pollen type grains germinate on *papillose* stigmas and B-pollen type grains germinate in *cob*-like stigmas, while complementary combinations do not produce successful fertilization (Baker 1953a, b; Richards 1997). In *L. ovalifolium,* meiocytes generally follow a regular course of meiosis leading to the formation of A- and B type pollen grains, likely involved in the production of seeds. Since in most populations analyzed, both pollen and stigma types are present (data not shown), cross fertilization may be favored. Nevertheless, since we identified a significant frequency of seeds with triploid embryos, they could be derived from unreduced male gametes, as most populations produce low frequencies of large grains. Alternatively, we cannot exclude that low levels of facultative apomixis could have occurred in the individuals studied. In *Limonium* triploids, *Ixeris*-type embryo sacs with non-reduced gametes have been observed (D’Amato 1949). In the case of the *Limonium* tetraploids here studied, although the present dataset do not prove the hypothesis of apomixis, they tend to suggest that at least pollen-independent endosperm formation takes place in this system due to the observed poor pollen quality. As reported in other model systems, poor quality of pollen due to mutation accumulation (Muller’s ratchet) relaxes selection for a male function and will result in many of the progeny sired by asexuals being weak or inviable (Mogie et al. 2007). Detailed female sporogenesis and gametogenesis and seed development studies would clarify whether or not apomictic phenotypes are produced.

## Supplementary data

Supplementary data consists of the following files. Two files containing histograms from cell nuclei of bulked seed samples of *Limonium ovalifolium* (Fig. S1) and of *L. multiflorum* (Fig. S2) obtained with a Partec PAII flow cytometer.

## Electronic supplementary material

Below is the link to the electronic supplementary material.

Supplementary material 1 (TIFF 101 kb)

Supplementary material 2 (TIFF 161 kb)

## References

[CR1] Arrigoni PV, Diana S (1993). Contribution à la connaissance du genre *Limonium* en Corse. Candollea.

[CR2] Artelari R (1989). Biosystematic study of the genus *Limonium* (*Plumbaginaceae*) in the Aegean area (Greece). I. Some *Limonium* species from the Kikladhes islands. Willdenowia.

[CR3] Artelari R, Georgiou O (2002). Biosystematic study of the genus *Limonium* (*Plumbaginaceae*) in the Aegean area, Greece. EO. *Limonium* on the islands Kithira and Antikithira and the surrounding islets. Nord J Bot.

[CR4] Baker HG (1948). Dimorphism and monomorphism in the *Plumbaginaceae*: I A survey of the family. Ann Bot.

[CR5] Baker HG (1953). Dimorphism and monomorphism in the *Plumbaginaceae*: II. Pollen and stigmata in the genus *Limonium*. Ann Bot.

[CR6] Baker HG (1953). Dimorphism and monomorphism in the *Plumbaginaceae*: III. Correlation of geographical distribution patterns with dimorphism and monomorphism in *Limonium*. Ann Bot.

[CR7] Baker HG (1966). The evolution, functioning and breakdown of heteromorphic incompatibility systems. I. The Plumbaginaceae. Evolution.

[CR102] Bhatt AM, Canales C, Dickinson HG (2001) Plant meiosis: the means to 1N. Trends Plant Sci 6:114–121. doi:10.1016/S1360-1385(00)01861-6 10.1016/s1360-1385(00)01861-6 11239610

[CR8] Brehm J, Maxted N, Ford-Lloyd BV, Martins-Loução A (2008). National inventories of crop wild relatives and wild harvested plants: case study for Portugal. Genet Resour Crop Evol.

[CR9] Brullo S, Pavone P (1981). Chromosome numbers in the Sicilian species of *Limonium* Miller (*Plumbaginaceae*). An Jard Bot Madr.

[CR10] Caperta AD, Rosa M, Delgado M, Karimi R, Demidov D, Viegas W, Houben A (2008). Distribution patterns of phosphorylated Thr 3 and Thr 32 of histone H3 in plant mitosis and meiosis. Cytogenet Gen Res.

[CR11] Castro M, Rosseló JA (2007). Karyology of *Limonium* (*Plumbaginaceae*) species from the Balearic Islands and the western Iberian Peninsula. Bot J Linn Soc.

[CR12] Cosendai A-C, Hörandl E (2010). Cytotype stability, facultative apomixis and geographical parthenogenesis in *Ranunculus kuepferi* (*Ranunculaceae*). Ann Bot.

[CR13] Cowan R, Ingrouille MJ, Lledó MD (1998). The taxonomic treatment of agamosperms in the genus *Limonium* Mill. (*Plumbaginaceae*). Folia Geobot.

[CR100] D'Amato F (1940) Contributo all'embriologia delle *Plumbaginaceae*. Nuovo Giorn Bot Ital 47(2):349–382

[CR14] D’Amato F (1949). Triploidia e apomissia in *Statice oleaefolia* Scop. var. *confusa* Godr. Caryologia.

[CR15] Dahlgren KVO (1916). Zytologische und embryologische Studien uber die Reihen Primulales und Plumbaginales. Kongligen Svenska Vetenskaps Akademiens Handlingar.

[CR16] Dawson HJ, Ingrouille MJ (1995). A biometric survey of *Limonium vulgare* Miller and *L. humile* Miller in the British Isles. Watsonia.

[CR17] Diana S (1995). Variabilità cariologica in *Limonium bonifaciense* Arrigoni et Diana (*Plumbaginaceae*). Bol Soc Sarda Sci Nat.

[CR18] Dolcher T, Pignatti S (1967). Numeri cromosomici di alcune specie mediterranee del genere *Limonium*. Nuovo Giornale Botanico Italiano.

[CR19] Dolcher T, Pignatti S (1971). Un’ipotesi sull’evoluzione dei *Limonium* del bacino mediterraneo. Nuovo Giornale Botanico Italiano.

[CR20] Erben M (1978). Die Gattung *Limonium* im südwestmediterranen Raum. Mitteilungen der Botanischen Staatssammlung München.

[CR21] Erben M (1979). Karyotype differentiation and its consequences in Mediterranean *Limonium*. Webbia.

[CR22] Erben M (1993) *Limonium* Mill. In: Castroviejo S, Aedo C, Cirujano S, Lainz M, Montserrat P, Morales R, Garmendia FM, Navarro C, Paiva J, Soriano C (eds.) Flora Iberica 3:2–143

[CR23] Erben M (1999). *Limonium nydeggeri*—eine neue Ar taus Sudwestportugal. Sendtnera.

[CR24] Erdtman G (1952) Pollen morphology and plant taxonomy, Angiosperms. The Chronica Botanica Co., Walthan, Mass. Printed by Almquist Wiksell, Stockholm, Sweden

[CR101] Galbraith DW, Harkins KR, Maddox JM, Ayres NM, Sharma DP, Firoozabady E (1983) Rapid flow cytometric analysis of the cell cycle in intact plant tissues. Science 220:1049–1051 10.1126/science.220.4601.1049 17754551

[CR25] Greuter W, McNeill J, Barrie FR, Burdet HM, Demoulin V, Filgueiras S, Nicolson DH, Silva PC, Skog JE, Trehane P, Turland NJ, Hawksworth DL (eds) (2000) International code of botanical nomenclature (Saint Louis Code). Regnum Vegetabile 138. Koeltz Scientific Books, 61453 Königstein, Germany. 2000. ISSN 0080-0694. ISBN 3-904144-22-7

[CR26] Hayat MA (1981). Principles and technics of electron microscopy: biological applications.

[CR27] Heslop-Harrison J, Heslop-Harrison Y (1970). Evaluation of pollen viability by enzymatically induced fluorescence: intracellular hydrolysis of fluorescein diacetate. Stain Tech.

[CR28] Ingrouille MJ (1984). A taxometric analysis of *Limonium* (*Plumbaginaceae*) in western Europe. Plant Syst Evol.

[CR29] Johansen DA (1940). Plant microtechnique.

[CR30] Kantama K, Sharbel TF, Schranz ME, Mitchell-Olds T, de Vries S, de Jong H (2007). Diploid apomicts of the *Boechera holboellii* complex display large-scale chromosome substitutions and aberrant chromosomes. PNAS.

[CR31] Karis PO (2004). Taxonomy, phylogeny and biogeography of *Limonium* sect. *Pteroclados* (*Plumbaginaceae*), based on morphological data. Bot J Linn Soc.

[CR32] Lledó MD, Crespo MB, Fay MF, Chase MW (2005). Molecular phylogenetics of *Limonium* and related genera (*Plumbaginaceae*): biogeographical and systematic implications. Am J Bot.

[CR33] Matzk F, Meister A, Schubert I (2000). An efficient screen for reproductive pathways using mature seeds of monocots and dicots. Plant J.

[CR34] Maynard-Smith J (1978). The evolution of sex.

[CR35] McCue AD, Cresti M, Feijó JA, Slotkin RK (2011). Cytoplasmic connection of sperm cells to the pollen vegetative cell nucleus: potential roles of the male germ unit revisited. J Exp Bot.

[CR36] Mogie M, Britton NF, Stewart-Cox (2007) Asexuality, polyploidy and the male function. In: Hörandl E, Grossniklaus U, Sharbel T F (eds): apomixis: evolution, mechanisms and perspectives. Regnum Veg. 147 A. R. G. Gantner Verlag

[CR37] Nowicke JW, Skvarla JJ (1977). Pollen morphology and the relationship of the *Plumbaginaceae*, *Polygonaceae*, and *Primulaceae* to the Order *Centrospermae*. Smithson Contrib Bot.

[CR38] Owen HA, Makaroff CA (1995). Ultrastructure of microsporogenesis and microgametogenesis in *Arabidopsis thaliana* (L.) Haynh. ecotype Wassilewskija (*Brassicaceae*). Protoplasma.

[CR39] Palacios C, Rosselló JA, González-Candelas F (2000). Study of the evolutionary relationships among *Limonium* species (*Plumbaginaceae*) using nuclear and cytoplasmic molecular markers. Mol Phyl Evol.

[CR40] Pignatti S (1971) Studi sui *Limonium*, VIII. In: Heywood VH (ed.) *Florae Europaea*. *Notulae systematicae ad flora Europaeam spectantes*: n° 11 Bot J Lin Soc 64:353–381

[CR41] Pinillos V, Cuevas J (2008). Standardization of the fluorochromatic reaction test to assess pollen viability. Biotech Histochem.

[CR42] Richards AJ (1997). Plant breeding systems.

[CR43] Ruzin SE (1999). Plant microtechnique and microscopy.

[CR44] Van Baarlen P, van Dijk PJ, Hoekstra RF, de Jong JH (2000). Meiotic recombination in sexual diploid and apomictic triploid dandelions (*Taraxacum officinale*). Genome.

[CR45] Voigt M-L, Melzer M, Rutten T, Mitchell-Olds T, Sharbel TF (2007) Gametogenesis in the apomictic Boechera holboellii complex: the male perspective. In: Hörandl E, Grossniklaus U, Sharbel T F (eds) Apomixis: evolution, mechanisms and perspectives. Regnum Veg. 147 A. R. G. Gantner Verlag

[CR46] Zhang C, Fountain WD, Morgan ER (1997). In vitro germination of the trinucleate pollen of *Limonium perezii*. Grana.

