# Supplementary figures and images for: Male fertility versus sterility, cytotype, and DNA quantitative variation in seed production in diploid and tetraploid sea lavenders (Limonium sp., Plumbaginaceae) reveal diversity in reproduction modes
Source: Sex Plant Reprod. 2012 Oct 20;25(4):305–18. doi: 10.1007/s00497-012-0199-y (PMC3493662; doi:10.1007/s00497-012-0199-y)

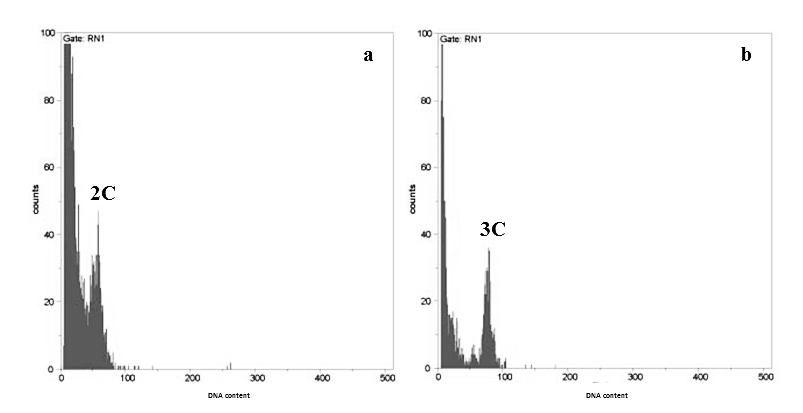

Supplement: Supplementary file 1 — Supplementary material 1 (TIFF 101 kb) [file 497_2012_199_MOESM1_ESM.tif]

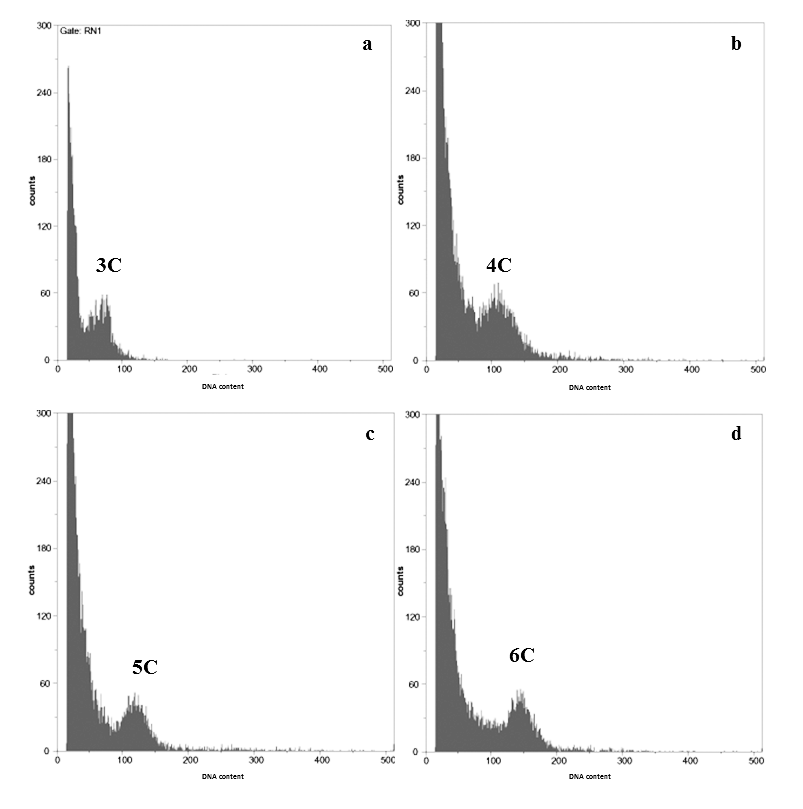

Supplement: Supplementary file 2 — Supplementary material 2 (TIFF 161 kb) [file 497_2012_199_MOESM2_ESM.tif]
